# Progress in transforming a health sciences postgraduate cohort in a south african research-intensive institution, 2008–2017

**DOI:** 10.1186/s12909-023-04691-6

**Published:** 2023-10-03

**Authors:** Moraba Meela, Beverley Kramer, Elena Libhaber

**Affiliations:** 1 School of Anatomical Sciences, Faculty of Health Sciences, University of the Witwatersrand, Johannesburg, South Africa; 2 Health Sciences Research Office, Faculty of Health Sciences, University of the Witwatersrand, Johannesburg, South Africa; 3 School of Clinical Medicine, Faculty of Health Sciences, University of the Witwatersrand, Johannesburg, South Africa

**Keywords:** Transformation, Postgraduate students, Higher education, Health sector, Population affinity, Sex

## Abstract

**Background:**

Equity redress in the higher education and health sectors is a global discourse that seeks to address the inequalities caused by past discrimination practices. The apartheid regime in South Africa fragmented both the higher education and the health sectors, creating White and male dominated systems. Consequently, Black Africans and females were under-represented in these sectors. Furthermore, the provision of higher education including medical training was unequal between the different populations. As democracy was established in South Africa in 1994, it is necessary to assess whether transformation in population affinity and sex of postgraduate students in the higher education and health sector has occurred, as these individuals are crucial for providing the future academic workforce and also healthcare to the public.

**Methods:**

The demographic profile of postgraduate students graduating in a health sciences facility in South Africa over the period 2008–2017 was retrospectively assessed. Survival analysis models were used to investigate the time taken to graduate. Log-rank tests were used to compare the completion rates.

**Results:**

More females (53.3%) than males (41.9%) completed their postgraduate degree over the period 2008–2017 (p˂0.0001). In relation to population affinity, more White students (56.4%) than Black African students (40.8%) completed their degrees overall (p˂0.0001).

**Conclusion:**

While transformation occurred in the sex of graduating students over the ten year period, the same change has not occurred with regards to population affinity. The under-representation of Black African graduates is a major setback for efforts to diversify the South African higher education and health sectors. Transformation of the demographic profile of postgraduate students at South African institutions is vital for developing individuals who will contribute to equitable redress of academic staff in the higher education sector and also of the healthcare workforce. Diversified health personnel including highly skilled clinician scientists will aid in improving the provision of health care to communities particularly the underpriviledged rural areas, and also assist in training the next generation of healthcare staff. The challenges identified in this study may assist other countries where adequate transformation of the education and health sectors has not occurred.

**Supplementary Information:**

The online version contains supplementary material available at 10.1186/s12909-023-04691-6.

## Background

The development of postgraduate students as a potential pool of academic staff for higher education institutions is important [1], but particularly so in underdeveloped countries. Furthermore postgraduate students graduating in a medical speciality are essential for the clinical fields of academia and the health system of a country, as they will provide specialised health care to communities. However, in South Africa the higher education system has a legacy of demographic discrimination emanating from the apartheid era of 1951, during which Black (including Coloured and Indian students) and White students were enrolled at separate academic institutions [2]. This created discrepancies in the provision of higher education, as institutions designated for White students only benefited from the largesse of the apartheid government, while those institutions to which Black students were assigned were inadequately resourced [3, 4].

This applied too, to the training medical students, as Black medical students were clinically trained at hospitals allocated for the Black population only [2, 5]. While medical schools for Black individuals had been set up, namely at MEDUNSA (currently called Sefako Makgatho Health Sciences University), the University of Natal (presently known as University of KwaZulu-Natal) and the University of Transkei (now called Walter Sisulu University), a limited number of Black medical doctors and allied healthcare workers were trained at postgraduate level [6, 7]. Lack of access to postgraduate medical training for Black individuals in South Africa created problems to the already over-burdened health system, as it could not meet the country’s health challenges.

Not only did the system of apartheid in South Africa create disparities between Black and White in the higher education and health sectors, but disproportions were also evident between the sexes. Females were generally excluded from both the higher education and the health sectors as apartheid created White and male dominated systems [7–9]. Black females were the most marginalised as they suffered from both the apartheid policies and from sexism [10, 11].

In 1994, when the new democratic government came into power in South Africa, there was consensus that both the higher education sector [12] and the health sector [6] needed to transform with respect to population affinity and sex. Accordingly, the Education White paper 3 was promulgated to address the inequalities created by apartheid in the higher education system [13]. In the health sector, the governing party’s health plan was adopted as the “post-apartheid model for health system change” [6, 14]. The plan was to tackle the demographic disparities in both the health sector and the unequal provision of healthcare to the population [6].

Despite interventions by the government, the process of transformation in both the higher education and the health sectors has been slow [6, 15, 16]. While increasing diversity in the configuration of student bodies in South Africa has occurred, the demographic profile of students is still not reflective of the population in which Black Africans constitute the majority [17, 18]. The disproportions in the demographics of students are said to be most evident at postgraduate level [16] which is the pipeline for future academic staff, and in health for healthcare personnel. If the disparities in the postgraduate student population in South African higher education institutions are not addressed, they will perpetuate the demographic imbalances of the past in both the academic and healthcare workforces.

The Wits Faculty of Health Sciences (FHS), which is one of the largest health sciences institutions in South Africa, services four major academic training hospitals, including the Chris Hani Baragwanath Hospital, the largest hospital in Africa. The Wits FHS trains both undergraduate and postgraduate students in medicine, dentistry, occupational therapy, physiotherapy, pharmacy, nursing, as well as science students majoring in the health sciences. Wits experienced the challenge of segregation during the apartheid era, as the Health Sciences Faculty was forced to use a quota system restricting Black student enrollments [2, 3]. Moreover, its departments such as dentistry, occupational therapy and physiotherapy were not allowed to admit Black students [2, 5]. While a dispensation was provided by the government to admit a limited number of Black African students into medicine, Black trainee doctors were not allowed to examine White patients and thus a dual system of training and patient care existed [2, 3, 5]. In light of this legacy of demographic inequalities and lack of access to higher education and clinical training facilities for previously disadvantaged population groups, Wits and the Wits FHS initiated a policy which addressed transformation issues [19]. In similar vein, Medical Schools in the United States of America (U.S.) also adopted affirmative action policies to redress past discrimination practices which prohibited minority groups particularly African-Americans and females from obtaining medical degrees [20].

The issue of transformation is not only a problem in South Africa but remains contentious in many parts of the world. The United Kingdom (UK) higher education sector is dominated by White and male academic staff and senior management [21]. While female academics in the UK are well represented in fields such as the arts and social sciences, education, health and community studies, and nursing and paramedical studies, White male academics still predominate in senior positions [21]. In the U.S. higher education system, the under-representation in academia of African-Americans, who were previously discriminated against, is an ongoing problem [22–24]. Furthermore, females in the U.S., particularly African-American females continue to be less represented at doctoral level although they graduate more frequently with bachelor’s degrees than do male students [25]. Thus, the attempts to transform the higher education sector in relation to sex and population affinity is a international phenomenon which appears to be far from being realised.

Compounding the issue of transformation is student completion of their postgraduate degrees. Student completion and non-completion (drop-out) rates are a serious concern for institutions of higher learning in South Africa [26, 27]. Reports have shown that students, particularly postgraduate students, may encounter barriers that prohibit them from obtaining their degrees, thus impeding transformation of the demographic profile of those graduating [28–30].

Thus, the aim of this study was to assess the number of graduations and the time taken to graduate of postgraduate students according to their demographics, over a ten year period (2008–2017) in a Faculty of Health Sciences based in a upper-middle income democratic country [31, 32] which has been undergoing transformation.

## Methods

### Permission and ethics

Permission to use the postgraduate student dataset was obtained from the Office of the Deputy Registrar at University of the Witwatersrand Johannesburg, South Africa. Ethics approval to conduct this study was obtained from the Wits Human Research Ethics Committee (Medical) [Clearance certificate number: M180262].

### Data source

Data for this study was obtained from the Business Intelligence Services (BIS), University of the Witwatersrand (Wits). The BIS manages all student data for the institution and updates records of Wits postgraduate students on an annual basis. The database is recorded as “Postgraduate Cohort Data”, anonymised, and stored in a secure file at the Wits BIS.

### Study population

All students registered for a postgraduate degree for the first-time in the Wits Faculty of Health Sciences (FHS) between 2008 and 2017 were considered for this study. Postgraduate students registered in the Wits Faculty of Science, but supervised by academics in the Wits FHS were also included. The binary male /female (sex) was used in this study as the Wits BIS was set up in this manner at the time of recording the data and did not contemplate the non-binary groupings [33] at the time of the development of the database. Population affinity was disaggregated into Black African, Chinese, Coloured, Indian and White as these terms are used in post-apartheid South Africa [34, 35].

Non-South African postgraduate students were excluded from the data set, as the current study focused on transformation of the South African population from a South African perspective as guided by the White Paper 3 of the current government of the Republic of South Africa [13].

### Study design

A retrospective review of the database of the Wits FHS was used to determine the number of graduations and time to qualification of postgraduate students according to their demographics over the ten year period (2008–2017). The variables were extracted from the database and transferred to an Excel worksheet. The following variables were included: age, date of graduation and graduation status, degree study mode: full-time/ part-time, population affinity, postgraduate degree for which the student was registered (Master’s by coursework – MC; Master of Dentistry – MDent; Master of Medicine – MMed; Master’s by research – MR; and Doctor of Philosophy – PhD), and sex. MC, MDent and MMed degrees consist of coursework and research while the MR and PhD are purely by research.

The duration of an enrolment at Wits for the MC and MR degrees is 2 years full-time and 3 years part-time according to the N + 1 rule which is implemented by Wits [36]. “N” is the minimum number of years allocated to finish a qualification. Thus according to the N + 1 rule, students are able to complete their degree in N + 1 years. The “+1” refers to the additional year that a student may need to finish a qualification [36]. The enrolment period for MDent and MMed is 4 years full-time and for the PhD, 3 years full-time and 5 years part-time in duration [36]. It is mandatory for dental and medical registrars who are specialising and training in Health Sciences Faculties in South Africa to undertake an MDent/MMed degree [36]. Students from other clinical disciplines such as Pharmacy and Occupational Therapy for example, may also be included in the Master’s degree by coursework.

### Statistical analysis

Analyses were conducted using Excel (Microsoft 2016) and Stata version 14.2 (StataCorp, College Station, TX). The 5 per cent level of statistical significance was used throughout.

Data were presented as frequencies and percentages.

The Chi square test was used to compare postgraduate student graduation proportions in 2008 and 2017.

For analysis purposes, the Chinese, Coloured and Indian students were combined into one category (Other) as their numbers were consistently small. Survival analysis models (Kaplan Meier plots and Fitting the Cox regression model) were used to investigate the time taken to graduate for a particular degree over the period 2008–2017, adjusted for population affinity and sex. Log-rank tests were used to compare the completion rates between the different population affinity groups, and between the sexes. For Kaplan Meier plots, the number of students considered (n = 4040) does not include the 186 students who dropped out in the first year.

## Results

A detailed breakdown of the sample with respect to sex, population affinity, degree type, and degree study mode (full-time/ part-time) is included in Supplementary Table 1.

### Relationship of sex or population affinity of the Wits FHS 2008–2017 cohorts of postgraduate students to the completion of their degree

Of the Wits FHS 2008–2017 cohort of postgraduate students in all degrees, a higher proportion of females (53.3%) than males (41.9%) completed their degree (p˂0.0001; Table 1). The overall proportion of both males and females who failed to complete their studies was 36% (Table 1). However, more males (45.15%) than females (31.36%) failed to complete their degree (Table 1). When the institutional designated time for completion of studies was considered, there was a similar rate of completion in the designated time between females and males (Table 1). Approximately 71% of both males and females took longer to complete their degrees than the institutional designated time, with more females than males taking longer to complete (Table 1).

**Table 1:** Outcome of degree attainment of Wits FHS postgraduate student classified according to sex over the period 2008–2017

Sex	N/ (%)	COT	CL	OOT	OL	FCD	Total registered
**Male**	**N**	160	444	31	156	651	1,442
	**%**	11.10	30.79	2.15	10.82	45.15	100.00
**Female**	**N**	367	1.117	75	352	873	2,784
	**%**	13.18	40.12	2.69	12.64	31.36	100.00
**Total**	**N**	527	1,561	106	508	1,524	4,226
	**%**	12.47	36.94	2.51	12.02	36.06	100.00

Pearson chi2 (4) = 79.5439 P<0.0001

Percentage (%) of rows

**COT** – completed on time (MC/ MR in 2 years full-time or 3 years part-time; MDent/ MMed 4 years full-time; PhD in 3 years full-time or 5 years part-time)

**CL** – completed late (i.e. not on time)

**OOT** – ongoing on time (MC/ MR in 2 years full-time or 3 years part-time; MDent/ MMed in 4 years full-time; PhD in 3 years full-time or 5 years part-time)

**OL** – ongoing late (so cannot complete on time/ in the institutional designated time)

**FCD** – Failed to complete the degree

With regards to population affinity, a higher percentage of White students (56.4%) than Black African students (40.8%) completed their degree overall (p˂0.0001; Table 2). Moreover, a higher proportion of White students (16.84%) than Black Africans (6.97%) completed their studies in the institutional designated time compared to the other categories of students. More Black African students (43.64%) than White students (29.96%) failed to complete their studies (Table 2).

**Table 2:** Outcome of degree attainment of Wits FHS postgraduate students classified according to population affinity over the period 2008–2017

Population affinity	N/ (%)	COT	CL	OOT	OL	FCD	Total Registered
**Black African**	**N**	114	553	44	211	714	1,636
	**%**	6.97	33.80	2.69	12.90	43.64	100.00
**Other**	**N**	150	390	28	118	342	1,028
	**%**	14.59	37.94	2.72	11.48	33.27	100.00
**White**	**N**	263	618	34	179	468	1,562
	**%**	16.84	39.56	2.18	11.46	29.96	100.00
**Total**	**N**	527	1,561	106	508	1,524	4,226
	**%**	12.47	36.94	2.51	12.02	36.06	100.00

Pearson chi2 (8) = 122.0755 P<0.0001

Percentage (%) of rows

**COT** – completed on time (MC/ MR in 2 years full-time or 3 years part-time; MDent/ MMed 4 years full-time; PhD in 3 years full-time or 5 years part-time)

**CL** – completed late (i.e. not on time)

**OOT** – ongoing on time (MC/ MR in 2 years full-time or 3 years part-time; MDent/ MMed 4 years full-time; PhD in 3 years full-time or 5 years part-time)

**OL** – ongoing late (so cannot complete on time/ in the institutional designated time)

**FCD** – Failed to complete the degree

### Graduation rates of the Wits FHS 2008–2017 cohorts of postgraduate students according to sex and population affinity

**Fig. 1:**
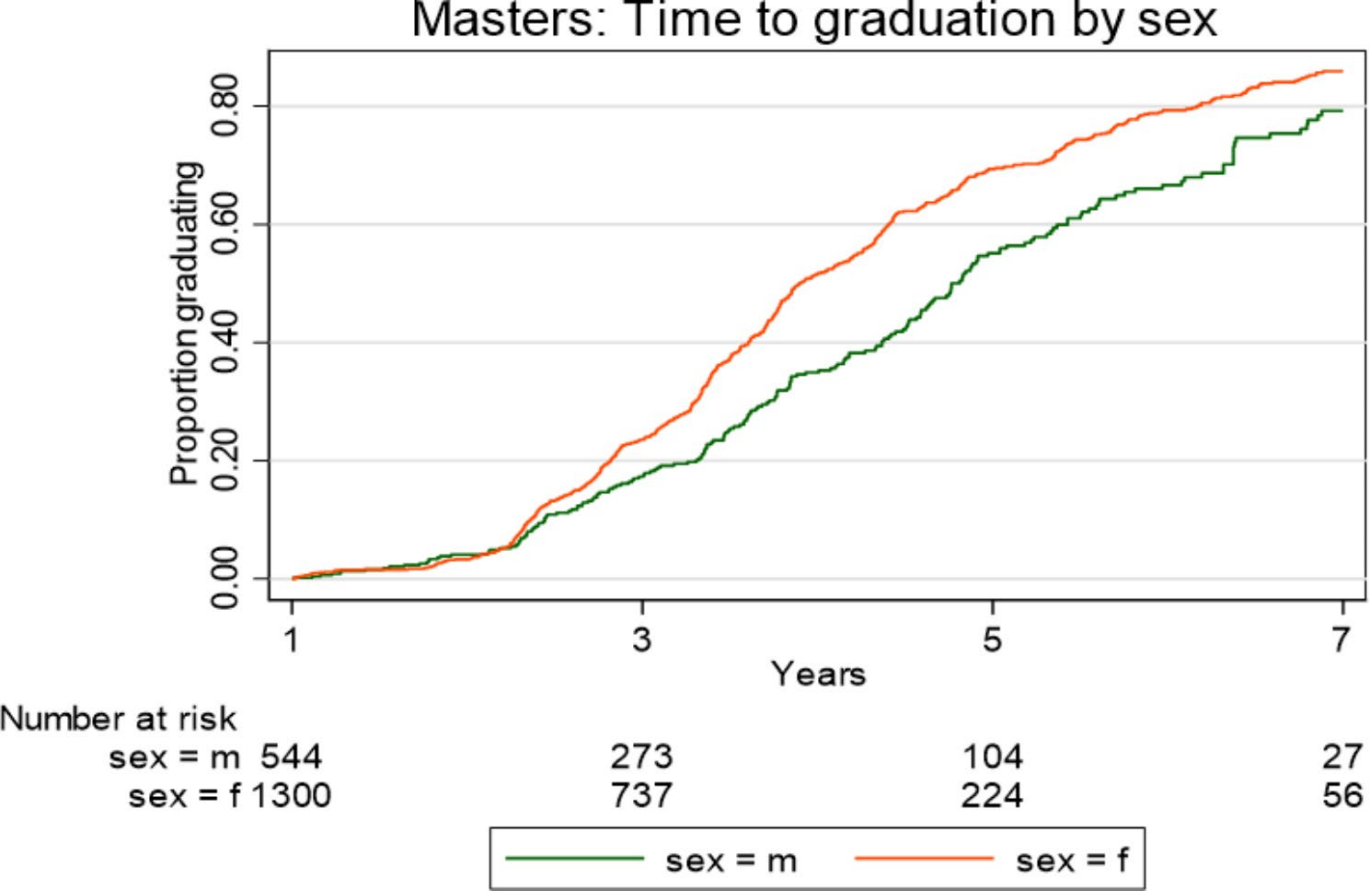
Time taken to graduate for Wits FHS postgraduate students at Master’s level (excluding MMed and MDent) according to sex over the period 2008–2017

**Fig. 2:**
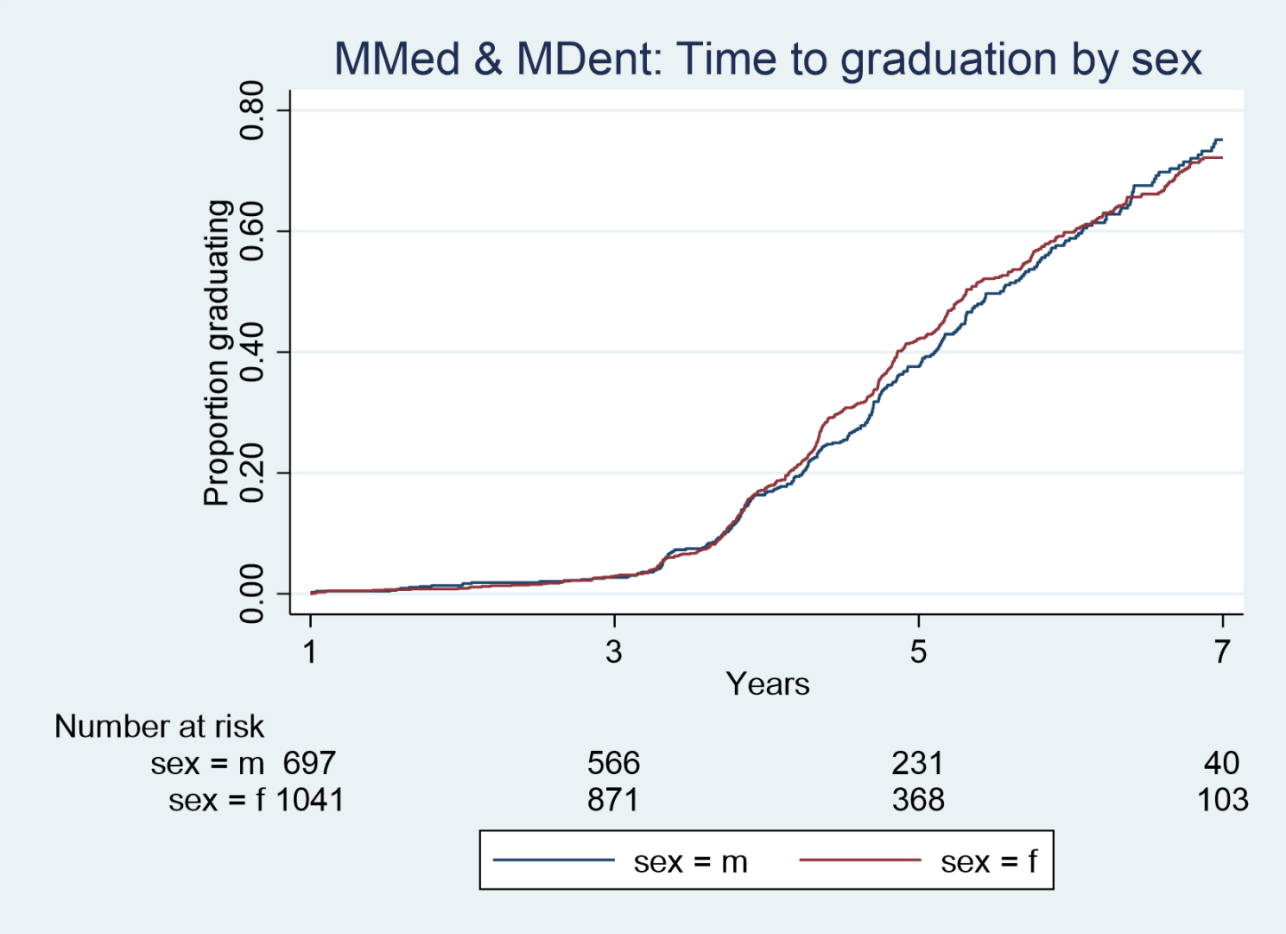
Time taken to graduate for Wits FHS MMed and MDent students according to sex over the period 2008–2017

**Fig. 3:**
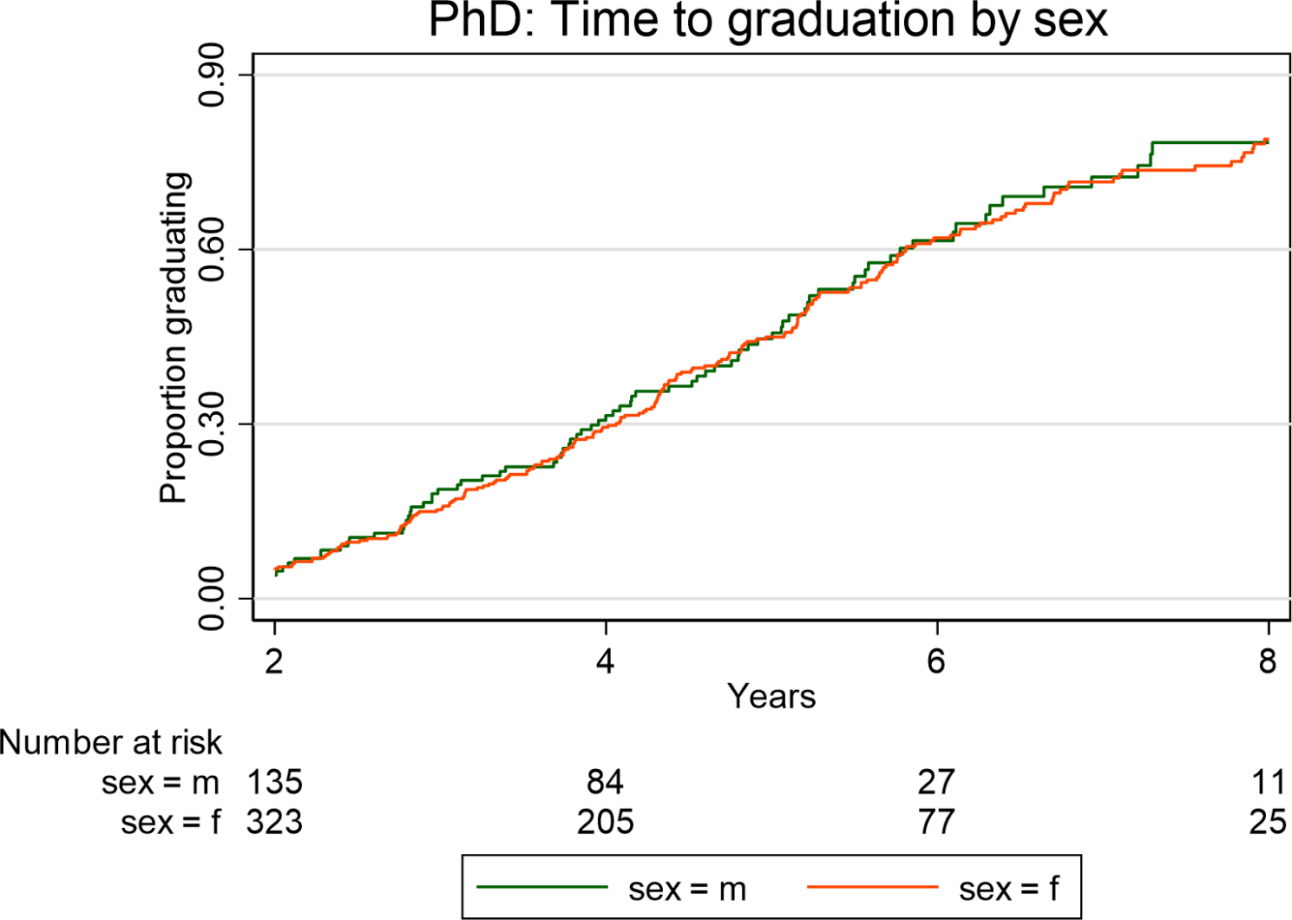
Time taken to graduate for Wits FHS South African postgraduate students at PhD level according to sex over the period 2008–2017

**Fig. 4:**
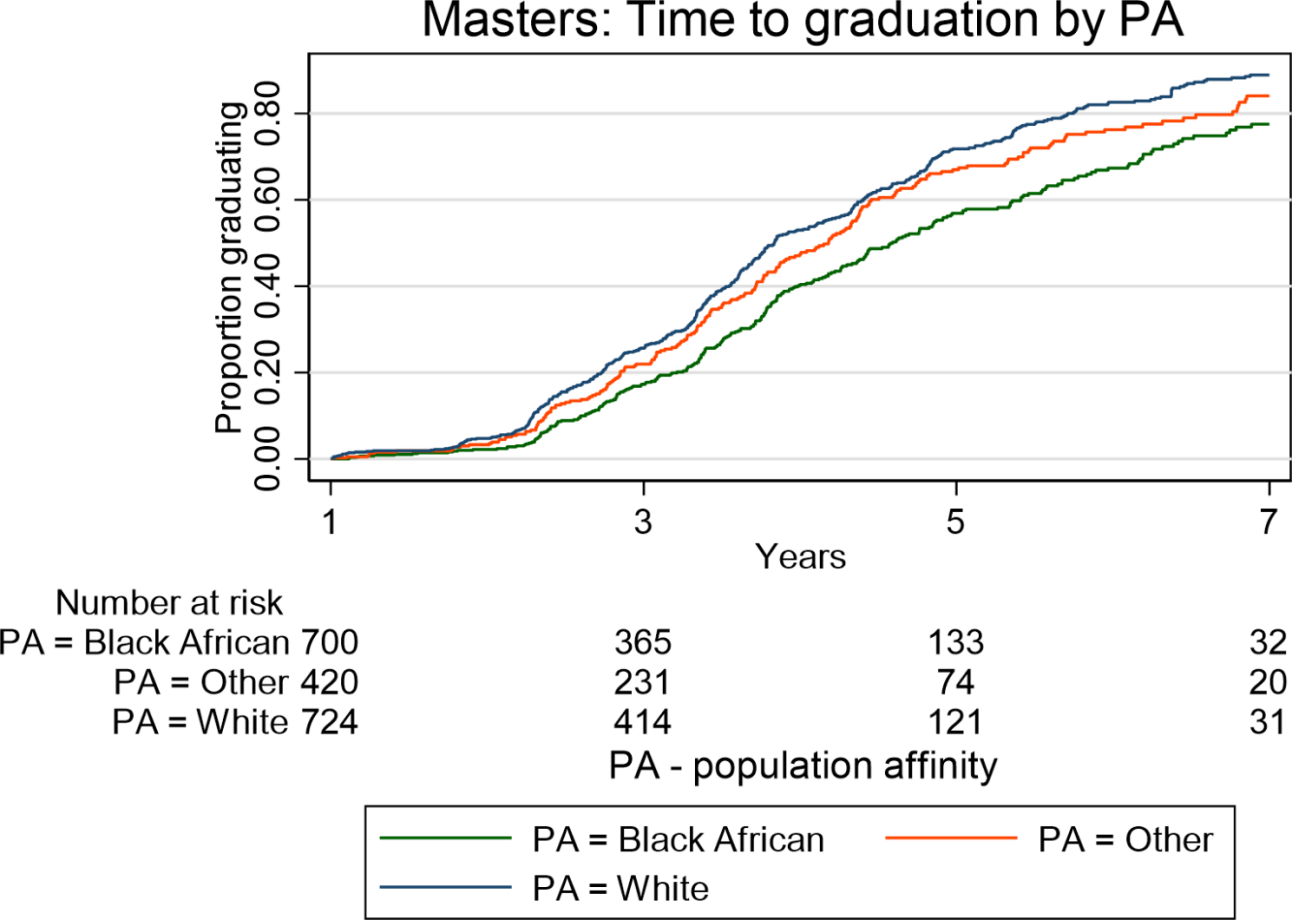
Time taken to graduate for the Wits FHS postgraduate students at Master’s level (excluding MMed and MDent) according to population affinity over the period 2008–2017

Kaplan Meier plots (Figs. 1, 2, 3 and 4) illustrate the time taken by a cohort of students to graduate for a particular degree in the Wits FHS. Those students who fail to complete at later years are included in the data until such time as they drop out.

More females than males graduated at Master’s level (MC and MR) over the period 2008–2017, p<0.0001 (Fig. 1). Females graduated faster than males at the MC and MR level (Fig. 1). However, in relation to MMed and MDent there were no statistically significant differences between female and male graduations in numbers or in time (Fig. 2). The time taken to graduate for females and males at MMed and MDent level was similar. Females and males also took a similar time to graduate at PhD level (Fig. 3).

**Fig. 5:**
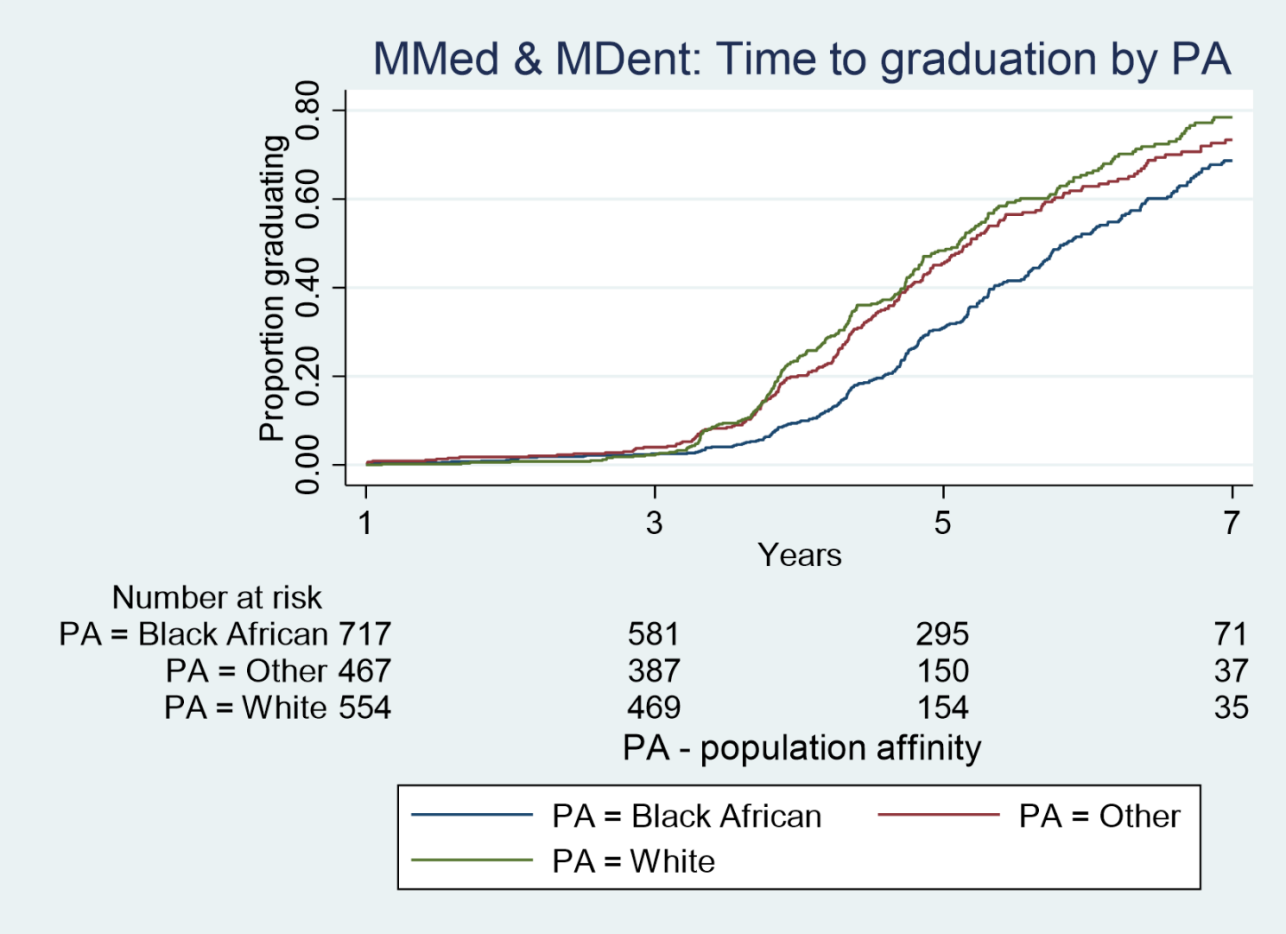
Time taken to graduate for the Wits FHS MMed and MDent students according to population affinity over the period 2008–2017

**Fig. 6:**
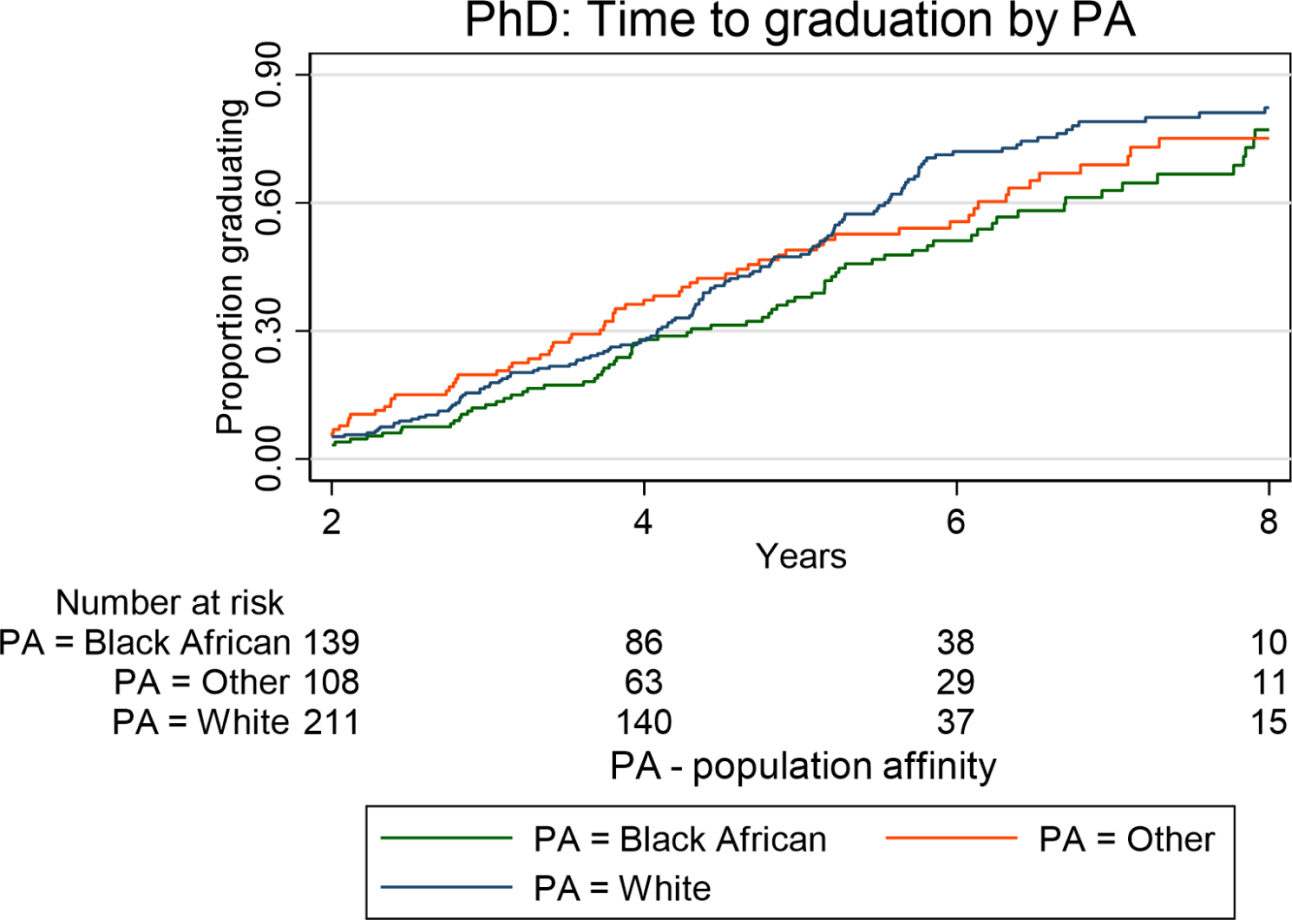
Time taken to graduate for the Wits FHS postgraduate students at PhD level according to population affinity over the period 2008–2017

In relation to population affinity, more White postgraduate students followed by students in the category “Other” graduated than did Black African students at Master’s level (MC and MR) (p < 0.0001; Fig. 4) over the period 2008–2017. Similarly, White graduations were slightly higher than “Other”, with Black African graduates being the lowest for graduation from the MMed and MDent degrees (p < 0.0001; Fig. 5). At PhD level, there were no statistically significant differences in the graduation rate or proportions between cohorts of students of different population affinities (Fig. 6).

### The rates of the Wits FHS postgraduate student graduations by demographics and type of degree during the period 2008–2017

At Master’s level (MC and MR), more females [16.4% (95%CI = 15.3–17.6, p < 0.0001)] than males [12.1% (95%CI = 10.6–13.8, p < 0.0001)] graduated (Table 3). Thus, for each 100 enrolled students per year, 16.4% of females graduated compared to 12.1% of males. Regarding population affinity, more White students [17.8% (95%CI = 16.2–19.5, p < 0.0001)] graduated per year than the other population groups, with Black African students [11.9% (95%CI = 10.6–13.4, p < 0.0001)] recording the lowest graduation rate. As expected, students who were enrolled full-time graduated in less time than did part-time enrolled students.

**Table 3:** Rates of postgraduate graduations by demographics and degree study mode (full-time or part-time) at Master’s level – MC and MR (excluding MMed and MDent)

Factor	Level	Number	Graduations	Person years (pyar)	Graduation rate per 100 pyar (95% CI)	Logrank X^2^; P-value
Overall		1910	956 (50.0%)	6310.0	15.2 (14.2 ; 16.1)	
Full time / part time	Full time Part time	679 1231	385 (56.7%) 571 (46.4%)	2212.1 4097.9	17.4 (15.8 ; 19.2) 13.9 (12.8 ; 15.1)	X^2^_(1)_ = 23.0 P < 0.0001
Sex	Male Female	567 1343	218 (38.4%) 738 (55.0%)	1808.2 4501.8	12.1 (10.6 ; 13.8) 16.4 (15.3 ; 17.6)	X^2^_(1)_ = 20.36 P < 0.0001
Population Affinity	Black African Other White	732 430 748	279 (38.1%) 229 (53.3%) 448 (59.9%)	2342.7 1442.2 2523.1	11.9 (10.6 ; 13.4) 15.9 (13.9 ; 18.1) 17.8 (16.2 ; 19.5)	X^2^_(2)_ = 30.44 P < 0.0001

For the MMed and MDent degrees, female and male graduation rates per year were not significantly different (Table 4). In relation to population affinity, more White students [11.8% (95%CI = 10.5–13.3, p < 0.0001)] graduated with a MMed or MDent degree per year compared to Black African students [9.1% (95%CI = 8.1–10.2, p < 0.0001)].

**Table 4:** Rates of postgraduate graduations by demographics and degree study mode for MMed and MDent

Factor	Level	Number	Graduations	Person years (pyar)	Graduation rate per 100 pyar (95% CI)	Logrank X^2^; P-value
Overall		1780	828 (46.5%)	7843.5	10.6 (9.9 ; 11.3)	
Sex	Male Female	714 1780	297(41.6%) 531 (49.8%)	3037.2 4806.4	9.8 (8.7 ; 11.0) 11.0(10.1 ; 12.0)	X^2^_(1)_ = 0.25 P = 0.62
Population Affinity	Black African Other White	741 475 564	306 (41.3%) 238 (50.1%) 284 (50.4%)	3358.4 2078.4 2406.7	9.1 (8.1 ; 10.2) 11.5 (10.1 ; 13.0) 11.8 (10.5 ; 13.3)	X^2^_(2)_ = 36.4 P < 0.0001

At PhD level, there were no significant differences between female and male graduation rates (Table 5). Similarly, individuals of different population affinity groups did not differ significantly in their graduation rates.

**Table 5:** Rates of postgraduate graduations by demographics and degree study mode (full-time or part-time) at PhD level

Factor	Level	Number	Graduations	Person years (pyar)	Graduation rate per 100 pyar (95% CI)	Logrank X^2^; P-value
Overall		536	304 (56.7%)	2339.3	13.0 (11.6 ; 14.5)	
Full time / part time	Full time Part time	279 257	186 (66.7%) 118 (45.9%)	1141.0 1198.3	16.3 (14.1 ; 18.8) 9.8 (8.2 ; 11.8)	X^2^_(1)_ = 32.9 P < 0.0001
Sex	Male Female	161 375	89 (55.3%) 215 (57.3%)	686.0 1653.0	13.0 (10.5 ; 16.0) 13.0 (11.4 ; 14.9)	X^2^_(1)_ = 0.03 P = 0.86
Population Affinity	Black African Other White	163 123 250	82 (50.3%) 73 (59.4%) 149 (59.6%)	729.7 545.2 1064.4	11.2 (9.0 ; 14.0) 13.4 (10.6 ; 16.8) 14.0 (11.9 ; 16.4)	X^2^_(3)_ = 3.91 P = 0.14

### Analysis of the rate of postgraduates graduating – fitting the cox regression model for time to graduation

The adjusted Hazard ratio provides the likelihood of a Wits FHS postgraduate student graduating, given that one has not yet graduated. Thus, a White student in the FHS is 1.46 times (HR, 1.46; 95%CI, 1.32–1.62; p < 0.001) more likely to graduate than a Black African student in the FHS at any given point in time in relation to the years analysed here (Table 6). Females in the FHS are also 1.14 times more likely to graduate than males, and part-time students (HR, 0.77; 95%CI, 0.61–0.81; p < 0.001) are less likely to graduate in the prescribed time compared to full-time students.

**Table 6:** Fitting the Cox regression model for time to graduation

Factor	Level	Adjusted HR (95% CI)	LR P-value
Full time / part time	Full Time Part Time	1 (reference) 0.77 (0.61 ; 0.81)	< 0.001
Sex	Male Female	1 (reference) 1.14 (1.04 ; 1.25)	0.007
Population Affinity	Black African Other White	1 (reference) 1.28 (1.14 ; 1.43) 1.46 (1.32 ; 1.62)	< 0.001

## Discussion

In the current study undertaken on health sciences postgraduate students in a South African institution between 2008 and 2017, female students dominated graduations and more White than Black African students completed their degrees.

Male graduates had previously predominated in South African higher education institutions across all areas of study, resulting in more male graduates at Master’s (55%) and PhD (56 − 58%) level [15]. In the U.S. and Iceland, similar to the findings of the current study, more females than males graduate with postgraduate qualifications [37, 38]. A separate global study of the scientific fields concurs that females dominate graduations at postgraduate level, and account for 53% of graduates at Master’s level in 2013 [39]. Recently, at the graduation ceremonies hosted by the University of KwaZulu-Natal, South Africa, 65% of graduates in both undergraduate and postgraduate courses were women [40].

The overall high percentages of graduations of female postgraduate students in the present study indicates transformation of the Wits FHS graduates in terms of the sexes. This is crucial for equity redress in the higher education and the health systems in South Africa as females were previously disadvantaged and under-represented in all spheres of society during the apartheid era [8, 11, 41]. Female graduates worldwide dominate in all postgraduate degree types, although their numbers decrease abruptly at PhD level [38, 39]. Despite the increasing number of females graduating with postgraduate degrees, males in the U.S. are still over-represented in many employment areas which pay high salary packages. Thus, as in South Africa, transformation with respect to sex of the individual is still an ongoing process in both the higher education and the workplace for certain countries [20–22].

Although female graduates in general predominated in the Wits FHS during the period 2008–2017, the graduation rates of females and males were similar in the Master of Medicine and Master of Dentistry degrees. These degrees are the prerequisite degrees for specialization in medicine and dentistry respectively, in South Africa. A diversified healthcare workforce is good for social integration and may assist in tackling the country’s complex health challenges [42, 43]. Participation of females in the health sector will bring diverse ideas and views on challenges in the health sciences [44] and may entice more women to join the profession, as female postgraduates may choose a female mentor or role model, in recognition of the understanding of their mutual challenges [45–48].

The equivalent female and male completion rates of PhD graduates in the Wits FHS is mirrored in other sub-Saharan Africa studies where the graduation rate of females and males in science, technology, engineering, and mathematics PhD programmes are similar [29, 49]. Although the present study indicates parity of female and male Wits FHS PhD graduates, more females than males graduated in the Master’s by coursework and Master’s by research which may indicate a “leaking” postgraduate pipeline with respect to females. In two separate studies conducted globally [39] and in sub-Saharan Africa [29], the representation of female students decreased gradually as students proceeded up the education and career hierarchy owing to various factors during postgraduate training. Parenting, getting married and family responsibilities are some of the factors that prohibit females from graduating with a PhD [28, 29]. Interventions such as family orientated policies that adequately support women’s roles as wives and mothers, mentoring and supervisory support among other factors, should be implemented by higher education institutions in order to increase the representation of females with PhD degrees and in senior career positions [26, 29].

The continuing predominance of White postgraduate graduates in the Wits FHS is of concern for the future. The change to democracy in South Africa which led to a conversion in the demographics of students attending institutions of higher learning occurred almost 30 years ago, yet an increase in Black African postgraduate graduations has not occurred. Black Africans constitute the majority of the South African population [17] compared to the other population groups. Therefore, transforming the population affinity of graduates is central to assisting in diversifying the academic workforce and in health sciences particularly, for the healthcare workforce which can aid in tackling the burden of diseases and the existing problems of a strained South African health system [50, 51].

Postgraduate research capacity development and particularly, the production of clinician scientists [52, 53] is crucial for health research. South Africa like the rest of Africa, suffers from a huge burden of disease, and in order to tackle this appropriately, clinicians who have undertaken training in research are needed to lead clinically-related research [52]. The relatively low rates of MMed/MDent graduates completing their degrees does not currently support the production of clinician scientists. In 2011, the Health Professions Council of South Africa (HPCSA) made the research component of the degree of specialization mandatory, and this may improve the number of graduates graduating with these degrees in the future [54, 55].

The high rates of Black African postgraduate students who fail to complete their degrees in the Wits FHS is a cause for concern, as equitable graduate outputs appear to be far from being realised. Furthermore, postgraduate students who fail to complete their degrees are costing the South African government billions in grants and subsidies to higher education institutions without return on investment [56]. Herman [30] reported on the Department of Higher Education estimation that “a student drop-out rate of 20% implies that about 1.3 billion in government subsidies is spent each year on students who do not complete their study programme”. Some of the known reasons for not completing their degrees (Masters and PhD) in the Wits FHS, include transfer of these postgraduate students to the Wits undergraduate medical programme, a change of career path or of institution, a change of medical speciality, other employment opportunities and lack of funding (Upublished survey data). Family commitments i.e. being married or having children, inadequate exposure to research, language barriers, shortage of training courses and work responsibilities are some of the other factors cited both locally and globally [26, 57, 58].

Similarly, family or personal responsibilities were reported as the key barriers affecting South African older and part-time postgraduate students taking longer to graduate [27, 30]. While some of the Wits FHS postgraduate students in the current study also take longer to complete their studies, the causes for the delay have not yet been investigated. Recommedations for reducing the drop-out rates of postgraduate students are providing funding tailored for the circumtances of undepriviledged students, particularly Black Africans, improving education at school level to produce quality students and a change in institutional culture to accommodate students from diverse backgrounds [59].

### Limitations of the study

Transformation in relation to individuals with disabilities was not considered as this is beyond the scope of the present study.

## Conclusion

Overall, more female than male postgraduate students graduated and more White than Black African students completed their degrees over the period 2008–2017 in a South African Health Sciences institution. Thus, while transformation in graduations of this institution’s postgraduate student body has occurred in terms of sex, it did not occur in relation to population affinity. The under-representation of Black African graduates is a serious drawback for efforts to transform the South African higher education and health sectors, as postgraduate students are a potential pool of future academic and health workforces. Transforming the demographic profile of the Wits FHS graduates at postgraduate level is important for building capacity which will contribute to equity redress of academic staff, and in the case of the health sector, to diversify the healthcare workforce so that it can assist in the improvement of patient care especially to the underserved rural communities. Further analysis into barriers that impede Black African postgraduate students from graduating is being undertaken.

### Electronic supplementary material

Below is the link to the electronic supplementary material.

**Supplementary Table 1**: Outcome of degree attainment of Wits FHS postgraduate students classified according to sex, population affinity, degree type, and degree study mode (full-time/ part-time) over the period 2008 ? 2017

## Data Availability

The data that support the findings of this study are available from the Wits, Business Intelligence Services but restrictions apply to the availability of these data, which were used under license for the current study, and so are not publicly available. Data are however available from the corresponding author upon reasonable request and with permission of the Wits, Business Intelligence Services.

## List of abbreviations

BIS: Business Intelligence Services
COT: Completed on time
CL: Completed late
FCD: Failed to complete the degree
FHS: Faculty of Health Sciences
MC: Master’s by coursework
MDent: Master of Dentistry
MMed: Master of Medicine
MR: Master’s by research
OL: Ongoing late
OOT: Ongoing on time
PhD: Doctor of Philosophy
U.S.: United States of America
UK: United Kingdom
Wits: University of the Witwatersrand
