# Supplementary material for: Progress in transforming a health sciences postgraduate cohort in a south african research-intensive institution, 2008–2017
Source: BMC Med Educ. 2023 Oct 3;23:721. doi: 10.1186/s12909-023-04691-6 (PMC10548610; doi:10.1186/s12909-023-04691-6)
Supplement: Supplementary file 1 — Supplementary Table 1: Outcome of degree attainment of Wits FHS postgraduate students classified according to sex, population affinity, degree type, and degree study mode (full-time/ part-time) over the period 2008 ? 2017 [file 12909_2023_4691_MOESM1_ESM.docx]

**Supplementary Table 1:** Outcome of degree attainment of Wits FHS postgraduate students classified according to sex, population affinity, degree type, and degree study mode (full-time/ part-time) over the period 2008 – 2017

| **Sex and Population Affinity** | **N/ (%)** | **MC** | | **MC Total** | **MMed/**  **MDent** | **MMed/**  **MDent**  **Total** | **R** | | **R Total** | **PhD** | | **PhD Total** | **Total Graduated** | **Total registered** |
| --- | --- | --- | --- | --- | --- | --- | --- | --- | --- | --- | --- | --- | --- | --- |
| **Female** |  | Full-time | Part-time |  | Full-time |  | Full-time | Part-time |  | Full-time | Part-time |  |  |  |
| **Black African** | **N** | 24 | 93 | 117 | 195 | 195 | 67 | 13 | 80 | 32 | 19 | 51 | 443 | 1012 |
|  | **%** | 2,37 | 9,19 | 11,56 | 19,27 | 19,27 | 6,62 | 1,28 | 7,91 | 3,16 | 1,88 | 5,04 | 43,77 | 100 |
| **Other** | **N** | 17 | 77 | 94 | 162 | 162 | 62 | 26 | 88 | 38 | 18 | 56 | 400 | 688 |
|  | **%** | 2,47 | 11,19 | 13,66 | 23,55 | 23,55 | 9,01 | 3,78 | 12,79 | 5,52 | 2,62 | 8,14 | 58,14 | 100 |
| **White** | **N** | 41 | 170 | 211 | 174 | 174 | 77 | 71 | 148 | 58 | 50 | 108 | 641 | 1084 |
|  | **%** | 3,78 | 15,68 | 19,46 | 16,05 | 16,05 | 7,10 | 6,55 | 13,65 | 5,35 | 4,61 | 9,96 | 59,13 | 100 |
| **Total Female** | **N** | 82 | 340 | 422 | 531 | 531 | 206 | 110 | 316 | 128 | 87 | 215 | 1484 | 2784 |
|  | **%** | 2,95 | 12,21 | 15,16 | 19,07 | 19,07 | 7,40 | 3,95 | 11,35 | 4,60 | 3,13 | 7,72 | 53,30 | 100 |
| **Male** |  | | | | | | | | | | | | | |
| **Black African** | **N** | 16 | 31 | 47 | 111 | 111 | 28 | 7 | 35 | 21 | 10 | 31 | 224 | 624 |
|  | **%** | 2,56 | 4,97 | 7,53 | 17,79 | 17,79 | 4,49 | 1,12 | 5,61 | 3,37 | 1,60 | 4,97 | 35,90 | 100 |
| **Other** | **N** | 2 | 26 | 28 | 76 | 76 | 14 | 5 | 19 | 10 | 7 | 17 | 140 | 340 |
|  | **%** | 0,59 | 7,65 | 8,24 | 22,35 | 22,35 | 4,12 | 1,47 | 5,59 | 2,94 | 2,06 | 5 | 41,18 | 100 |
| **White** | **N** | 11 | 44 | 55 | 110 | 110 | 26 | 8 | 34 | 27 | 14 | 41 | 240 | 478 |
|  | **%** | 2,30 | 9,21 | 11,51 | 23,01 | 23,01 | 5,44 | 1,67 | 7,11 | 5,65 | 2,93 | 8,58 | 50,21 | 100 |
| **Total Male** | **N** | 29 | 101 | 130 | 297 | 297 | 68 | 20 | 88 | 58 | 31 | 89 | 604 | 1442 |
|  | **%** | 2,01 | 7 | 9,02 | 20,60 | 20,60 | 4,72 | 1,39 | 6,10 | 4,02 | 2,15 | 6,17 | 41,89 | 100 |
| **Grand Total** | **N** | 111 | 441 | 552 | 828 | 828 | 274 | 130 | 404 | 186 | 118 | 304 | 2088 | 4226 |
|  | **%** | 2,63 | 10,44 | 13,06 | 19,59 | 19,59 | 6,48 | 3,08 | 9,56 | 4,40 | 2,79 | 7,19 | 49,41 | 100 |

**MC** – Master’s by coursework; **MDent** – Master of Dentistry; **MMed** – Master of Medicine; **MR** – Master’s by research; and **PhD** – Doctor of Philosophy
